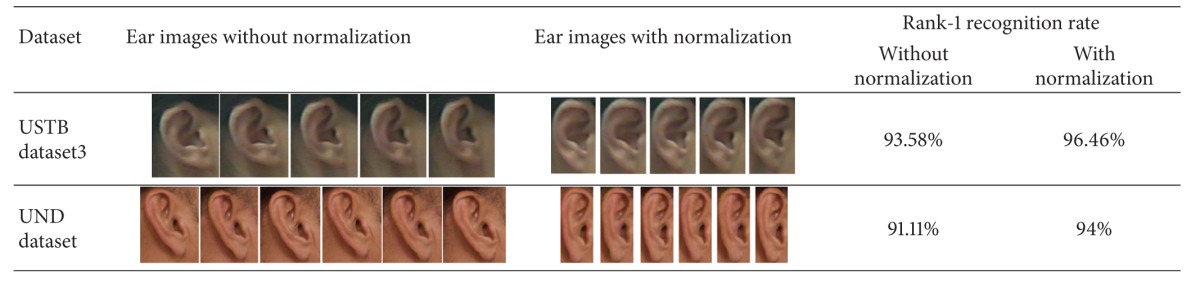# Ear Recognition Based on Gabor Features and KFDA

**DOI:** 10.1155/2014/702076

**Published:** 2014-03-17

**Authors:** Li Yuan, Zhichun Mu

**Affiliations:** ^1^School of Automation and Electrical Engineering, University of Science and Technology Beijing, Beijing, 100083, China; ^2^Visualization and Intelligent Systems Laboratory, University of California Riverside, Riverside, CA, 92507, USA

## Abstract

We propose an ear recognition system based on 2D ear images which includes three stages: ear enrollment, feature extraction, and ear recognition. Ear enrollment includes ear detection and ear normalization. The ear detection approach based on improved Adaboost algorithm detects the ear part under complex background using two steps: offline cascaded classifier training and online ear detection. Then Active Shape Model is applied to segment the ear part and normalize all the ear images to the same size. For its eminent characteristics in spatial local feature extraction and orientation selection, Gabor filter based ear feature extraction is presented in this paper. Kernel Fisher Discriminant Analysis (KFDA) is then applied for dimension reduction of the high-dimensional Gabor features. Finally distance based classifier is applied for ear recognition. Experimental results of ear recognition on two datasets (USTB and UND datasets) and the performance of the ear authentication system show the feasibility and effectiveness of the proposed approach.

## 1. Introduction

The research on ear recognition has been drawing more and more attention in recent five years [1–4]. Based on the research of the “Iannarelli system” [5], the structure of the ear is fairly stable and robust to changes in facial expressions or aging. Ear biometrics is noncontacting and so it can be applied for human identification at a distance, making it a helpful supplement to facial recognition. An ear recognition system based on 2D images is composed of the following stages: ear enrollment, feature extraction, and ear recognition/authentication. The stage of ear enrollment includes automatic ear detection and ear normalization. Ear detection focuses on detecting human ear from the input images and then locating and segmenting each ear in the image. Then all the ear images are normalized to the same size based on some predefined standard. Next step is to represent the ear by appropriate features, such as structural features, local features, and holistic features. Finally effective classifier will be designed for ear recognition or authentication, such as nearest neighbor classifier, sparse representation classifier, RBF classifier, or SVM classifier.

### 1.1. Related Work

Ear recognition using 2D images can be categorized into three kinds based on the features extracted from the ear images: (1) structural features, (2) local features, and (3) holistic features. The application scenarios can be summarized as the following three: ear recognition under constrained environment, ear recognition with pose variation, and ear recognition under partial occlusion.  Table 1 outlines some representative references for different feature extraction methods and their corresponding performance on different ear datasets.

### 1.2. Our Work

The present works listed in Table 1 mainly focused on feature extraction and classification. The ear images are mostly manually extracted for later processing. The alignment process was not clearly illustrated in most of the ear recognition papers. So the ear images used in different methods were not normalized based on the same standard. Therefore the comparisons among different methods are less meaningful. So [6, 7] proposed automatic ear detection based on Adaboost algorithm. The ear parts presented in source images are segmented. But these segmented ear images may contain not only the “pure ear,” but also some background image (such as face profile and hair). This means that even for the same subject, the size of each “pure ear” on the registered images in the dataset may be different, and it is also possible that the size of ears on the registered images is not the same as that of the ear to be authenticated. So many appearance based methods will not work in this situation. This means that there exists a gap between ear detection and feature extraction. This gap is automatic ear normalization, very much like face normalization, which means that we have to set up a standard to normalize the ear into the same size. In this paper, we combine ear detection and ear normalization into one stage named ear enrollment. To our best knowledge, the research on ear enrollment is still an open area.

The rest of this paper is organized as follows.  Section 2 describes our automated ear enrollment approach.  Section 3 details the feature extraction approach using Gabor filters and Kernel Fisher Discriminant Analysis.  Section 4 conducts ear recognition and ear authentication experiments to evaluate the proposed methods. Finally, concluding remarks are drawn in Section 5.

## 2. Ear Enrollment

This section will detail the ear enrollment process, which includes ear detection and ear normalization. The ear detection approach based on our modified Adaboost algorithm detects the ear part under complex background using two steps: offline cascaded classifier training and online ear detection. We have made some modification compared with our previous work on ear detection [6]. Then Active Shape Model is then applied to segment the ear part and normalize all the ear images to the same size.

### 2.1. Automatic Ear Detector Based on Modified Adaboost Algorithm

In our previous work [6], we have proposed an ear detection approach under complex background which has two stages: offline cascaded classifier training and online ear detection. The cascaded classifier is composed of multiple strong classifiers. Each strong classifier is composed of multiple weak classifiers. The training process of a strong classifier is as follows.

(1) Given *N* example images (*x*_1_, *y*_1_),…, (*x*_*n*_, *y*_*n*_), where  *x* ∈ *ℜ* ^*k*^, *y*_*i*_ ∈ {−1,1} for negative and positive examples, respectively.
(2) Initialize weights  *D*_1_(*x*_*i*_) = 1/*n*, *i* = 1,2,…, *n*.
(3) Repeat for *t* = 1,2,…, *T*; *T* is the total number of weak classifiers; do the following:
  (i) train the weak classifiers with weights *D*_*t*_, and get the regression function *h*_*t*_ : *X* → {−1, +1}. The nearest neighbor classifier is used as the weak classifier;
  (ii) get the error rate of the regression function *ε*_*t*_ = ∑_*i*=1_^*n*^ *D*_*t*_(*x*_*i*_)[*h*_*t*_(*x*_*i*_) ≠ *y*_*i*_];
  (iii) set the weight of weak classifiers *α*_*t*_: *a*_*t*_ = 1/2ln⁡((1 − *ε*_*t*_)/*ε*_*t*_);
  (iv) update weights of different training samples:
    (1) $$\begin{array}{c} D_{t+1}\left(x_{i}\right)=\frac{D_{t}\left(x_{i}\right)}{Z_{t}}\times \{\begin{array}{c} e^{-\alpha_{t}}\begin{array}{cc} & \text{if}\begin{array}{cc} & h_{t}\left(x_{i}\right)=y_{i} \end{array} \end{array}\\ e^{\alpha_{t}}\begin{array}{cc} & \text{if}\begin{array}{cc} & h_{t}\left(x_{i}\right)\neq y_{i}, \end{array} \end{array} \end{array} \end{array}$$
    where *Z*_*t*_ is the normalization factor for ∑_*i*=1_^*n*^ *D*_*t*_(*x*_*i*_) = 1.
(4) After *T* times of training, the final strong classifier is
  (2) $$\begin{array}{c} H\left(x\right)=\mathrm{sign}\left(\sum_{i=1}^{T}\alpha_{t}h_{t}\left(x\right)-\text{Th}\right), \end{array}$$
  where Th is the decision threshold decided by the false classification rate.

The training process of the algorithm mentioned above is time consuming. Also, the false rejection rate and false acceptance rate need to be lowered for real application scenarios. Based on the structural features of the ear itself, we propose the following four improvements on the traditional AdaBoost algorithm in view of its deficiency.

*Improvement 1*. In Adaboost algorithm, the selection of the optimum classification threshold of the Haar-like features is very important for weak classifier learning algorithm. This procedure is time consuming. So we propose a “segment-selecting method” to choose the optimum threshold of weak classifiers.

**Step 1**

We divide the feature value space composed of the feature values of all the training samples for each Haar-like feature into *n* parts and *r* feature values per part. Suppose that the original feature value space is *g*(*i*)  (*i* = 0,…, *N*), where *N* is the total number of positive and negative samples. The new feature value space is *g*(*k*)  (*k* = 0, *r*, 2*r*,…, *n*).

**Step 2**

In the new space, search the optimum threshold *g*(*j*). Then back to the original feature value space, centered with *g*(*j*), we search the region from *g*(*j* − *r*) to *g*(*j* + *r*) to find out the optimum threshold.

*Improvement 2*. We apply a strategy which can reduce the false acceptance rate, by means of changing the weight distribution of weak classifiers.

The strong classifier is composed of weighted weak classifiers. The smaller error rate a weak classifier possesses, the bigger the weight assigned to a weak classifier. The error rate is decided by the training samples. The positive and negative samples are equally important. Ear detection experimental results show that with the traditional Adaboost algorithm, the false acceptance rate is not acceptable. So we improve the training procedure of the weak classifiers by proposing that the weight distribution among the weak classifiers not only is decided by the total error rate, but also is concerned with the negative samples.

So we improve the Adaboost algorithm by including a parameter *ke* ^*q*_*t*_^ to give higher weights to those weak classifiers that have lower false acceptance rate on negative samples. *q*_*t*_ is the weight sum of those negative samples that have been classified correctly, representing the classification ability of the weak classifier to negative samples; *k* is used to constrain the impact of *q*_*t*_ on the weight of the weak classifier. The modified Adaboost algorithm is as follows.

**Step 1**

Given a weak classifier learning algorithm and a training sample set (*x*_1_, *y*_1_),…, (*x*_*n*_, *y*_*n*_), where *x*_*i*_ is the training sample feature vector, *y*_*i*_ ∈ {−1, +1} for negative and positive examples, respectively.

**Step 2**

Initialize the weights *D*_1_(*x*_*i*_) = 1/*n*, *i* = 1,2,…, *n*.

**Step 3**

Repeat for *t* = 1,2,…, *T*; *do* the following:

(1) train the weak classifier learning algorithm with the weights *D*_*t*_, and return the weak classifier *h*_*t*_ : *X* → {−1, +1};
(2) compute the error rate;
  (3) $$\begin{array}{c} \varepsilon_{t}=\sum_{i=1}^{n}D_{t}\left(x_{i}\right)\left[h_{t}\left(x_{i}\right)\neq y_{i}\right] \end{array}$$
  get the weight sum of those negative samples that are classified correctly as
  (4) $$\begin{array}{c} q_{t}=\sum_{i=1}^{n}D_{t}\left(x_{i}\right)\left[y_{i}=-1,h_{t}\left(x_{i}\right)=-1\right] \end{array}$$
(3) get the updating parameter *α*_*t*_ and the weight parameter of weak classifier *α*_*t*−new_:
  (5) $$\begin{array}{c} a_{t}=\frac{1}{2}\ln\left(\frac{1-\varepsilon_{t}}{\varepsilon_{t}}\right),\\ a_{t-\text{new}}=\frac{1}{2}\ln\left(\frac{1-\varepsilon_{t}}{\varepsilon_{t}}\right)+ke^{q_{t}}, \end{array}$$
  where *k* is a constant value. The value of *k* should guarantee that the new added weak classifier will reduce the upper boundary of the minimum error rate. In this paper, *k* is set to 0.018.
(4) Update the weights:
  (6) $$\begin{array}{c} D_{t+1}\left(x_{i}\right)=\frac{D_{t}\left(x_{i}\right)}{Z_{t}}\times \{\begin{array}{c} e^{-\alpha_{t}}\begin{array}{cc} & \text{if}h_{t}\left(x_{i}\right)=y_{i} \end{array}\\ e^{\alpha_{t}}\begin{array}{cc} & \text{if}h_{t}\left(x_{i}\right)\neq y_{i}, \end{array} \end{array} \end{array}$$
  where *Z*_*t*_ is a normalization factor chosen so that ∑_*i*=1_^*n*^ *D*_*t*_(*x*_*i*_) = 1.

**Step 4**

After *T* loops, output the strong classifier:

(7) $$\begin{array}{c} H\left(x\right)=\mathrm{sign}\left(\sum_{i=1}^{T}\alpha_{t-\text{new}}h_{t}\left(x\right)-\text{Th}\right), \end{array}$$

where Th is the threshold corresponding with the error rate.

*Improvement 3*. We apply a new parameter called elimination threshold to improve the robustness of the detector and prevent overfitting.

By analyzing human ear samples, we find that the global structure of human ears is similar: the shape the outer contour is almost oval; all human ears have similar shape of helix, antihelix, and concha. These similar global features are helpful for the training of an ear/non-ear classifier. But if we look into more details, we find that each ear has its unique features or measures on different ear components. The differences on details make the Adaboost based two-class classifier more difficult to construct. Here, we regard those ear samples that present special detail components as “difficult samples.” These samples will get more weights during the weak classifier learning process, because the weak classifier will always try to get them classified correctly. This will ultimately incur overfitting. In order to prevent overfitting, we apply a new parameter called “elimination threshold Hw” to improve the robustness of the ear detector. During the training process, the ear samples with weight greater than Hw will be eliminated.

*Improvement 4*.  We propose the “single-ear-detection strategy” in view of the asymmetry of left ear and right ear. This strategy trains the detector with the samples of single ears. After the training process, we get left ear detector and right ear detector, which are called “single-ear detector.” On an input image, both detectors are applied to locate the ear part. In our previous work, both left ears and right ears images are used for training. We call this “dual-ear detector.” Compared with the dual-ear detector, the single-ear detector can improve the detection performance.

Table 2 shows the experimental results on three ear image datasets. These datasets are the same as used in our previous work [6]. In Table 2, FRN means false rejection number, FRR means false rejection rate, FAN means false acceptance number, and FAR means false acceptance rate. In Figure 11, we compare the number of weak classifiers on each layer among the dual-ear detector in [6], dual-ear detector in this paper, and single-ear detector in this paper. As we can see from Figure 11, compared with dual-ear detector, the single-ear detector contains less number of weak classifiers on each layer. With less number of weak classifiers, the training time can be reduced to 50% (CPU: 3.0 GHz; RAM: 2 G; the number of positive training samples is 11000; the number of negative training samples is 5000; the sample size is 60∗120). The training time for the left ear detector, right ear detector, and dual-ear detector is about 111 hours, 100 hours, and 217 hours, respectively.

**Figure 1:**
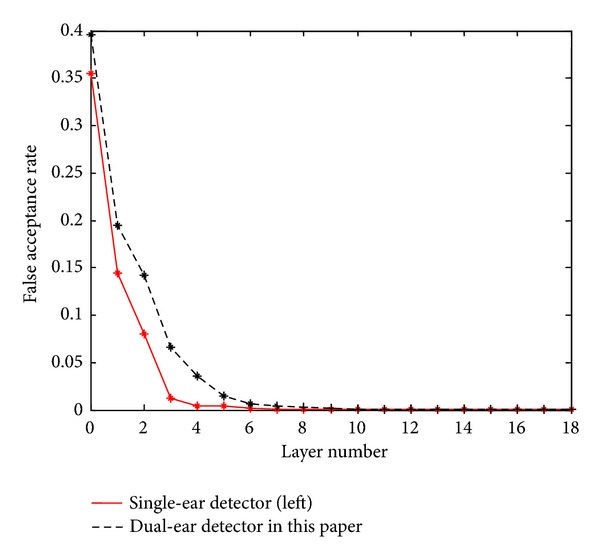
Performance comparison between single-ear detector and dual-ear detector.

**Figure 2:**
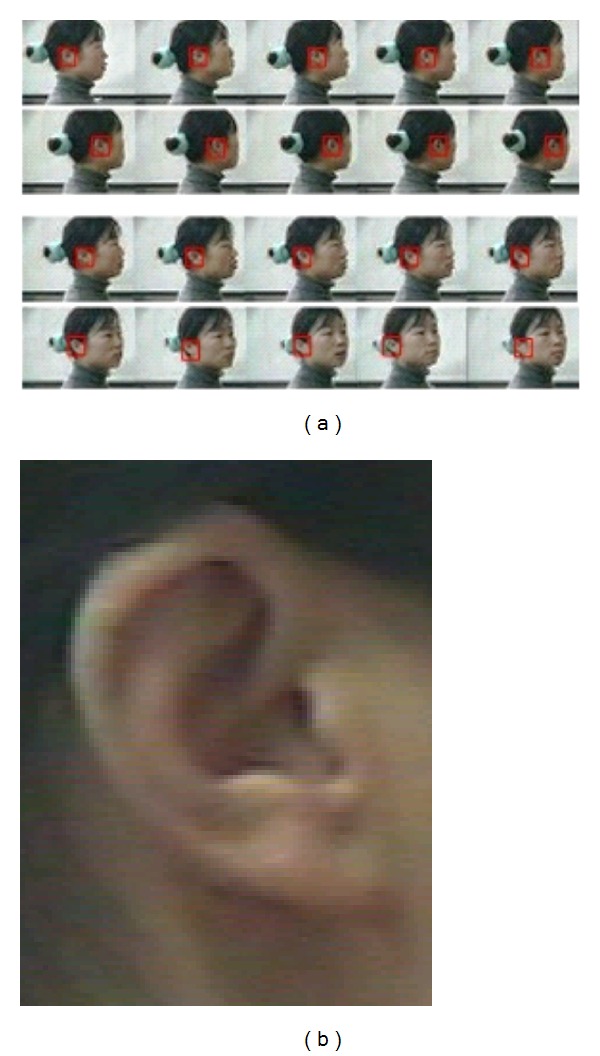
Ear detection experimental examples.

For each layer, we compare the detection performance between the single-ear detector and the dual-ear detector in this paper. For each layer, the difference of detection rate between these two detectors is very limited: not more than 1%, but they have difference on the false acceptance rate as shown in Figure 1; the single-ear detector has lower false acceptance rate, which means that it performs better on the negative sample.  Figure 2(a) shows an example of detection results on the registered images of one subject.  Figure 2(b) shows the extracted ear part.

### 2.2. Ear Normalization Using Active Shape Model

As we can see from the second figure in Figure 2(b), the segmented image contains some background information other than the pure ear, so we need to further extract the pure ear for the followed ear feature extraction.

**Figure 3:**
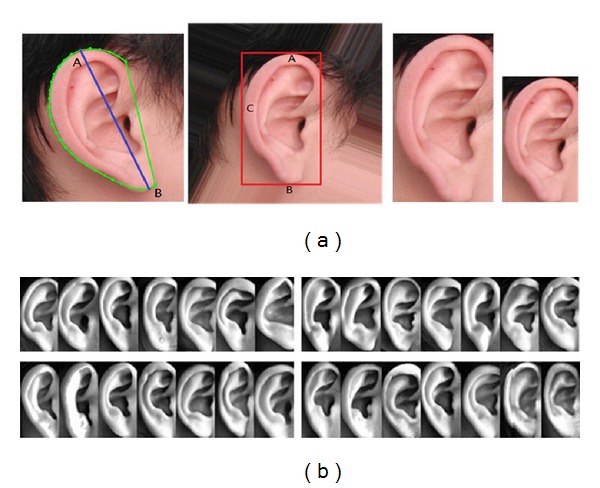
Example images of ear normalization.

We apply an automatic ear normalization method based on improved Active Shape Model (ASM) [8]. In the offline training step, ASM was used to create the Point Distributed Model using the landmark points on the ear images of the training set. This model was then applied on the test ear images to search the outer ear contour. The final step was to normalize the ear image to standard size and direction according to the long axis of outer ear contour. The long axis was defined as the line crossing through the two points which have the longest distance on the ear contour. After normalization, the long axes of different ear images will be normalized to the same length and same direction. This process is shown in Figure 3(a). The size of the normalized image is 60∗117 pixels. This ratio of 1.95 is set based on statistical research.

After converting the color images to gray images, we use histogram equalization to eliminate lighting variations among different subjects.  Figure 3(b) shows some example images after ear normalization.

## 3. Ear Recognition Based on Gabor Features

For feature extraction, Gabor filter is applied on the ear images to extract spatial local features of different directions and scales. The Gabor features are of high dimension, so Kernel Fisher Discriminant Analysis is further applied for dimension reduction. Then distance based classifier is applied for ear recognition.

### 3.1. Gabor Feature Extraction

The ear has its distinct features compared with the face, such as the texture features on different orientations. For its eminent characteristics in spatial local feature exaction and orientation selection, Gabor based ear is presented in this paper. A two-dimensional Gabor function is defined as [9]

(8) $$\begin{array}{c} g\left(x,y\right)=\exp\left(-\frac{x^{\prime^{2}}+y^{\prime^{2}}}{2\sigma^{2}}\right)\cos\left(2\pi \frac{x^{\prime }}{\lambda }+\phi \right),\\ \left[\begin{array}{c} x^{\prime }\\ y^{\prime } \end{array}\right]=\left[\begin{array}{cc} \cos\theta & \sin\theta \\ -\sin\theta & \cos\theta \end{array}\right]\left[\begin{array}{c} x\\ y \end{array}\right],\\ \frac{\sigma }{\lambda }=\frac{1}{\pi }\sqrt{\frac{\ln2}{2}}\frac{2^{b}+1}{2^{b}-1}. \end{array}$$

*λ* is the wavelength of the cosine factor of the Gabor filter kernel; its value is specified in pixels. *θ* specifies the orientation of the normal to the parallel stripes of a Gabor function. Its value is specified in degrees. *σ* is the standard deviation of the Gaussian factor. *b* is the half-response spatial frequency bandwidth.

**Figure 4:**
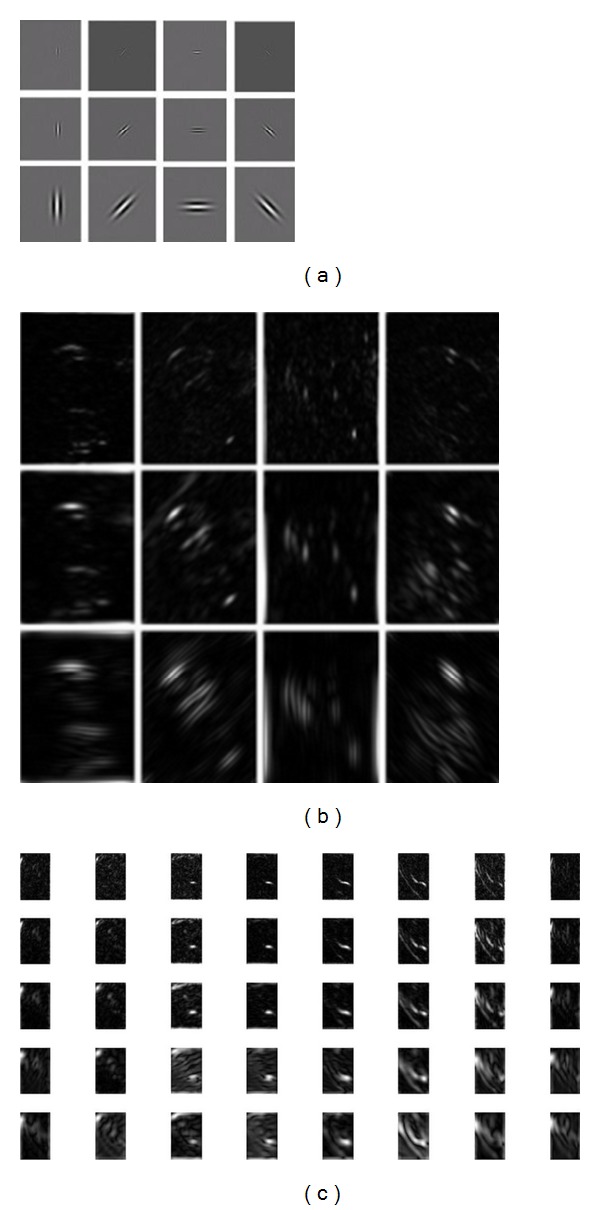
Gabor ear image: (a) the real part of the Gabor kernel; (b) the magnitude spectrum of the Gabor feature of the ear image on 4 orientations; (c) the magnitude spectrum of the Gabor feature of the ear image on 8 orientations.

The Gabor feature of an ear image is the convolution of the ear image and the Gabor kernel function as shown in

(9) $$\begin{array}{c} J\left(x,y,m,n\right)=\int I\left(x^{\prime },y^{\prime }\right)g_{mn}\left(x-x^{\prime },y-y^{\prime }\right)dx^{\prime }dy^{\prime }, \end{array}$$

where *m* and *n* stand for the number of scale and orientation of the Gabor wavelet. For each pixel (*x*, *y*), there are *m*∗*n* Gabor coefficients. Here we keep the magnitude of the Gabor filter as the ear feature.  Figure 4 shows the Gabor feature of the ear image.  Figure 4(a) shows the real part of the Gabor kernel on 3 scales (*λ* = 20, 25, and 30, resp., from the first to the third row, *θ* = 0, *π*/4, *π*/2, and 3*π*/4, resp., from the first to the fourth column).  Figure 4(b) shows the magnitude spectrum of the Gabor features corresponding to Figure 4(a), and 4(c) shows the magnitude spectrum of the Gabor features corresponding to 5 scales and 8 orientations. By this way, we can get the texture features on different orientations of the ear image. As we can see form Figure 4, the texture features of the human ear are salient enough on the four directions in Figure 4(b), so we choose these 4 orientations for future application in Section 3.2.

### 3.2. Dimension Reduction Using KFDA

For a *H*∗*W* pixels image, the dimension of the Gabor feature is *H*∗*W*∗*m*∗*n*. So the downsampling scheme is applied for dimension reduction of the high-dimensional Gabor features. Suppose the downsampling factor is *r*, and the Gabor coefficients are normalized to zero mean and unit variance. Then we concatenate all the coefficients *G*_*u*,*v*_^*r*^ to form the Gabor feature set *G* ^*r*^:

(10) $$\begin{array}{c} G^{r}={\left({\left(G_{0,0}^{r}\right)}^{t},{\left(G_{0,1}^{r}\right)}^{t}\cdots {\left(G_{2,3}^{r}\right)}^{t}\right)}^{t}. \end{array}$$

**Figure 5:**
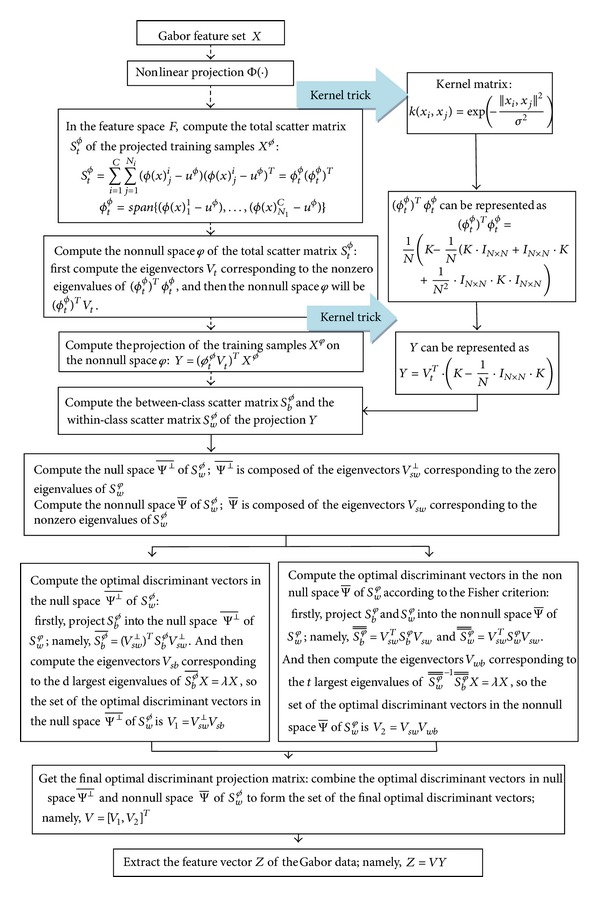
Dimension reduction using the Full Space Kernel Fisher Discriminant Analysis.

Suppose that *r* = 64; the feature dimension of an ear image with the size of 60∗117 will be 1317. The downsampling factor is empirically selected and set to 64 for all the experiments in this paper. So the feature dimension needs to be further reduced. In this paper, the Full Space Kernel Fisher Discriminant Analysis (FSKFDA) is used for feature reduction [10]. FSKFDA makes full use of the discriminant information in the full space of the within-class scatter, namely, the discriminant information in the null space and nonnull space of the within-class scatter.  Figure 5 shows the detailed flowchart of this method.

## 4. Experimental Results

### 4.1. Ear Recognition Experiment

**Figure 6:**
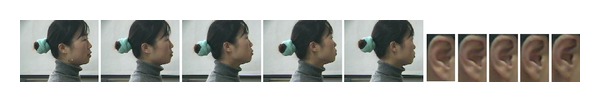
Sample images form USTB dataset3.

In this experiment, we select two datasets: the USTB dataset3 [11] and a subset of UND collection J2 [12]. The USTB dataset3 contains 79 subjects, and the images of each subject are taken under orientation variation. Five images for each subject are selected. These images are 10°, 5°of left rotation and 0°, 5°, and 10°of right rotation, respectively. Using the ear detection method mentioned in Section 2.1, we get the miss rate of 2/395 and the false alarm rate of zero on this USTB dataset3.  Figure 6 shows five source images and normalized ear images of one subject.

**Figure 7:**
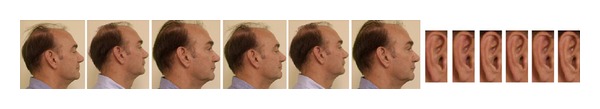
Sample images of UND dataset.

In UND collection J2, we selected 150 subjects, which possess more than 6 images per subject. Six images are selected for the experiment. There exists illumination and pose variation in this subset. Using the ear detection method mentioned in Section 2.1, we get the miss rate of 13/900 and the false alarm rate of 15/900 on this UND dataset.  Figure 7 shows six source images and normalized ear images of one subject.

In the training stage, we use the Gabor + KFDA method to get the feature space of the gallery set. In the test stage, the test image in the probe set is projected to the feature space to get its feature vector. Then the cosine distance measure based distance classifier is applied for classification. In (11), *x* and *y* represent two Gabor feature vectors:

(11) $$\begin{array}{c} \delta_{\cos}\left(x,y\right)=\frac{-x^{T}y}{||x||||y||}. \end{array}$$

For the kernel function in KFDA, we apply linear kernel, polynomial kernel, RBF kernel, and cosine kernel functions as shown in Table 3. We conduct a leave-one-out identification experiment. The averaged rank-1 recognition performance when adopting each kernel function is listed in Table 3 (the feature dimension is reduced to 100). The parameters in kernel functions are empirically selected in this paper. As we can see from Table 3, the recognition rate on both datasets performs best with the RBF kernel.

Table 4 compares the rank-1 recognition result using the RBF kernel in two scenarios: with ear normalization and without ear normalization. As we can see from Table 4, ear recognition with ear normalization performs better than ear recognition without ear recognition. We can see from the pictures in Table 4 that, in the “ear images without normalization” scenario, although the extracted ear images are set to the same size, the ear locations and the pure ear size may be different among these different enrolled images. On the other hand, in the “ear images with normalization” scenario, only the pure ear part is extracted and used for enrollment; all of the enrolled ears have the same size, which helps to enhance the recognition performance.

### 4.2. Ear Authentication Experiment

**Figure 8:**
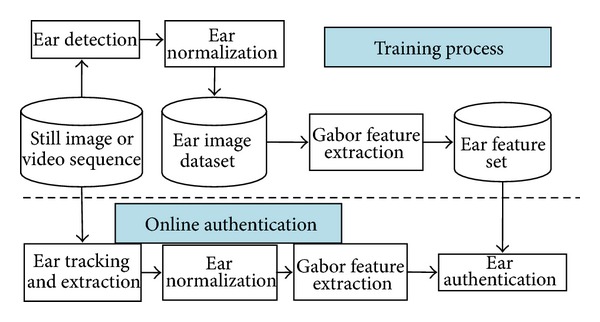
Framework flow of the ear authentication system.

We have designed an ear authentication system with the proposed method.  Figure 8 shows the framework flow of the ear authentication system.

**Figure 9:**
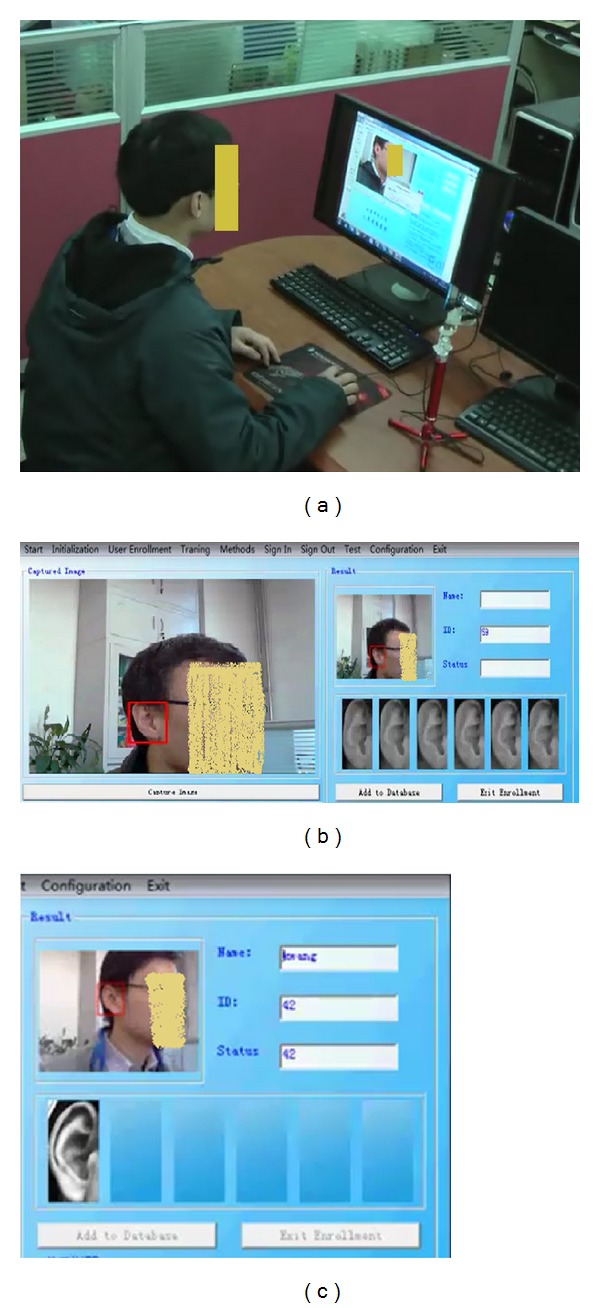
Ear authentication system: (a) authentication scenario; (b) ear enrollment interface; (c) ear authentication interface.

**Figure 10:**
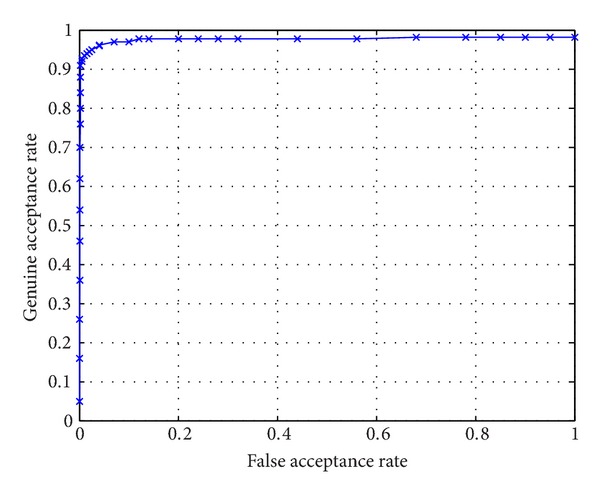
ROC curve of the ear authentication system.

**Figure 11:**
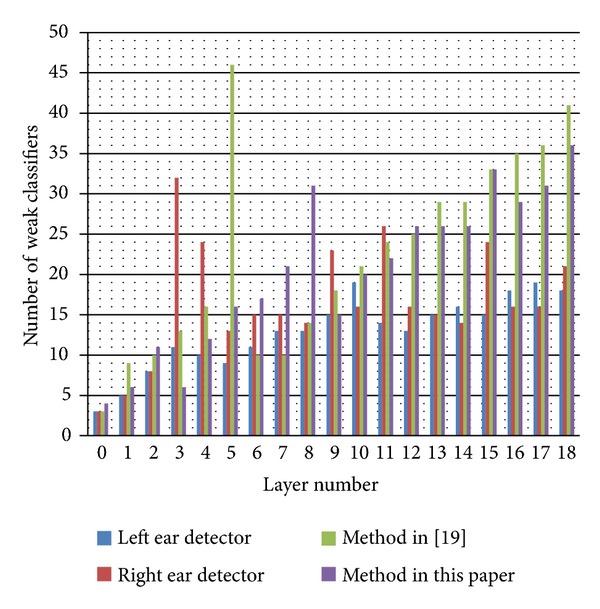
Comparison of the number of weak classifiers on each layer.

Figure 9(a) shows the application scenario. The system is composed of a camera (resolution is 640∗480) and a computer (CPU: i5 M520 2.40 GHz; RAM: 2.00 GB). There are a total number of 59 subjects registered. For each subject, 6 images are stored in the gallery set.  Figure 9(b) shows the ear enrollment process.  Figure 9(c) shows the ear authentication process. The time consumption for authenticating a subject is about 180 ms, in which the time consumption for the ear detector to extract the pure ear is about 150 ms.  Figure 10 shows the ROC curve of the authentication performance. The EER rate is about 4%. The experimental result shows that ear biometric is a feasible candidate for human identification.

## 5. Conclusion

In this paper, an ear recognition system based on 2D images is proposed. The main contributions of the proposed method are the following: (1) automatically detecting the ear part from the input image and normalizing the ear part based on the long axis of the outer ear contour and (2) extracting the discriminating Gabor features of the ear images using the Full Space Kernel Discriminant Analysis. Experimental results show that we can achieve automatic ear recognition based on 2D images with the proposed method.

Our future work will be focused on two aspects: (1) in the ear normalization stage, we need to improve the accuracy of the earlobe localization, generate deliberate models for the earlobe landmarks, and make the searching process less dependent on the initial model shape, and (2) in the ear authentication stage, we need a larger dataset to testify the matching accuracy and the real-time performance of the proposed method.

## Figures and Tables

**Table 1:** Representative feature extraction methods and their performance evaluation.

Reference	Description	Dataset	Performance
Structural feature extraction methods			

[13]	Burge and Burger (1997) use the main curve segments to form Voronoi diagram and use adjacency graph matching based algorithm for authentication. But the curve segments will be affected by changes in camera-to-ear orientation or lighting variation.	—	—

[14]	Moreno et al. (1999) used feature points of outer ear contour and information obtained from ear shape and wrinkles for ear recognition. The compression network is applied for classification.	28 subjects, 168 images, 6 images for each subject	Rank-1: 93%

[15]	Mu et al. (2004) proposed a long axis based shape and structural feature extraction method; the shape feature consisted of the curve fitting parameters of the outer ear contour, the structural feature was composed of ratios of the length of key sections to the length of the long axis, and nearest neighborhood classifier was used for recognition.	USTB dataset2: 77 subjects, 4 images for each subject	Rank-1: 85%

[16]	Choras (2005) proposed a geometrical feature extraction method based on number of pixels that have the same radius in a circle with the centre in the centroid and on the main curves.	240 images	—

Local feature extraction methods			

[17]	Hurley et al. (2005) proposed the force field transformation method. The ear images are treated as array of mutually attracting particles that act as the source of Gaussian force field. The force field transforms of the ear images were taken and the force fields were then converted to convergence fields. Then Fourier based cross-correlation techniques were used to perform multiplicative template matching on ternary thresholded convergence maps.	XM2VTS face profile subset (252 subjects)	Rank-1: 99.2%

[18]	Nanni and Lumini (2007) proposed a local approach. A multimatcher system was proposed where each matcher was trained using features extracted from the convolution of each subwindow with a bank of Gabor filters. The best matchers, corresponding to the most discriminative subwindows, were selected by Sequential Forward Floating Selection where the fitness function was related to the optimization of the ear recognition performance. Ear recognition was made using sum rule based decision level fusion.	UND collection E (114 subjects)	Rank-1: 80% EER: 4.3%

[19]	Bustard and Nixon (2010) proposed an ear registration and recognition method by treating the ear as a planar surface and creating a homography transform using SIFT feature matches. Ear recognition under partial occlusion was discussed in this paper. The relationship between occlusion percentage and recognition rate was presented.	XM2VTS face profile dataset (63 subjects)	Rank-1: 92% (30% occlusion from above), 92% (30% occlusion from left side)

[20]	Arbab-Zavar and Nixon (2011) proposed a model-based approach for ear recognition. The model was a partwise description of the ear derived by a stochastic clustering on a set of scale invariant features of the training set. The outer ear curves were further analyzed with log-Gabor filter. Ear recognition was made by fusing the model-based and outer ear metrics.	XM2VTS face profile dataset (63 subjects)	Rank-1: 89.4% (30% occlusion from above)

Holistic feature extraction methods			

[21]	Chang et al. (2003) used standard PCA to compare face and ear and concluded that ear and face did not have much difference on recognition performance.	Human ID Database (197 subjects)	Rank-1: 70.5% for face, 71.6% for ear

[22]	Yuan et al. (2006) proposed an improved Nonnegative Matrix Factorization with Sparseness Constraint for ear recognition with occlusion. The ear image was divided into three parts with no overlapping. INMFSC was applied for feature extraction. The final classification was based on a Gaussian model based classifier.	USTB dataset3 (79 subjects)	Rank 1: ~91% (for 10% occlusion from above)

[23]	Dun and Mu (2009) proposed an ICA based ear recognition method through nonlinear adaptive feature fusion. Firstly, two types of complimentary features are extracted using ICA. These features are then concatenated with different weight to form a high-dimensional fused feature. Then the feature dimension was reduced by Kernel PCA. The final decision was made by nearest neighbor classifier.	USTB dataset3 (79 subjects), and USTB dataset4 (150 subjects)	Rank-1: ≥90% (for pose variation within 15°)

[24]	Wang et al. (2008) proposed ear recognition based on Local Binary Pattern. Ear images were decomposed by Haar wavelet transform. Then Uniform LBP, combined with block-based and multiresolution methods, was applied to describe the texture features. Finally, the texture features are classified by the nearest neighbor method.	USTB dataset3 (79 subjects)	Rank-1: ≥92% (for pose variation within 20°)

[25]	Zhou et al. (2010) proposed ear recognition via sparse representation. Gabor features are used to develop a dictionary. Classification is performed by extracting features from the test data and using the dictionary for representing the test data. The class of the test data is then determined based upon the involvement of the dictionary entries in its representation.	UND G subset, 39 subjects	Rank-1: 98.46% (4 images for training and 2 images for testing)

**Table 2:** Detection performance comparison: FRR and FAR.

Test dataset	Number of ear images	Method in our previous work [6] (dual-ear detector)		Proposed method in this paper (dual-ear detector)		Proposed method in this paper (left ear detector + right ear detector)	
		FRN/FRR	FAN/FAR	FRN/FRR	FAN/FAR	FRN/FRR	FAN/FAR
CAS-PEAL	166	5/3.0%	6/3.6%	5/3.0%	2/1.2%	3/1.8%	2/1.2%
UMIST	48	1/2.1%	0/0%	0/0%	0/0%	1/2.1%	0/0%
USTB220	220	1/0.5%	5/2.3%	0/0%	5/2.3%	0/0%	1/0.5%

**Table 3:** Rank-1 recognition rate of the proposed method when adopting different kernel functions.

Kernel	*K*(*x*, *y*)	Parameters in kernel functions	Rank-1 recognition rate	
			USTB dataset 3	UND dataset
Linear	*K*(*x*, *x*_*i*_) = (*x* · *x*_*i*_)	—	75.44%	74.22%
Polynomial	*K*(*x*, *x*_*i*_) = [*a*(*x*·*x*_*i*_)+*b*]^*d*^	*a* = 0.001, *b* = 1, *d* = 2	89.11%	87.33%
RBF	*K*(*x*, *x*_*i*_) = exp⁡(−*γ*|*x*−*x*_*i*_|^2^)	*γ* = 0.0001	96.46%	94%
Cosine	$\tilde{k}(x,y)=\frac{k(x,y)}{\sqrt{k(x,x)k(y,y)}}$ *k*(*x*, *y*) = [*a*(*x*·*y*)+*b*]^*d*^	*a* = 0.001, *b* = 1, *d* = 2	92.41%	90.22%

**Table 4:** Performance comparison between ear recognition with or without ear normalization.